# UTILIZATION OF ROBOTIC ARM ASSISTANCE FOR REVISION OF PARTIAL TO TOTAL KNEE ARTHROPLASTY: A CASE REPORT

**DOI:** 10.1590/1413-785220263402e296465

**Published:** 2026-05-22

**Authors:** Eduardo Frois Temponi, Felipe Santiago de Almeida, Lúcio Honório de Carvalho, Matheus Braga Jacques Gonçalves, Ricardo Leão Carmo, Vitor Rodrigues Miranda

**Affiliations:** 1 Hospital Madre Teresa, Belo Horizonte, MG, Brazil.; 2 Pontifícia Universidade Católica, Belo Horizonte, MG, Brazil.; 3 Universidade Federal de Minas Gerais, Faculdade de Medicina Departamento de Medicina, Departamento do Aparelho Locomotor, Belo Horizonte, MG, Brazil.

**Keywords:** Review, Robotics, Arthroplasty, Total Knee, Arthroplasty, Partial Knee, Revisão, Robótica, Artroplastia do Joelho, Artroplastia Parcial do Joelho

## Abstract

**Introduction:**

The introduction of robotic-assisted total knee and hip arthroplasty in Brazil since 2021 has demonstrated benefits, including greater precision in bone cuts and reduced tissue trauma. However, there is a lack of studies on the use of this technology in TKA revisions. This report presents the first robot-assisted TKA performed in Brazil, representing an innovative and pioneering approach.

**Case report:**

A 61-year-old female patient presenting with pain and limited flexion in her left knee, who underwent unicompartmental knee arthroplasty (UKA) in 2015. After conservative treatment with physical therapy and injections proved ineffective, it was decided to proceed with robot-assisted total knee arthroplasty. The procedure followed the Mako protocol, which included robotic anatomical marking, removal of the previous components, and ligament adjustments. Precise bone cuts were made using the robotic arm, followed by the cementation of prosthetic components. The surgery went smoothly, with a favorable postoperative course.

**Conclusion:**

The use of robotic assistance in revision anterior cruciate ligament surgery has proven promising, yielding good postoperative results. However, further studies are needed to standardize the technique and establish specific protocols. ***Level of Evidence IV; Case Report.***

## RESUMO

**Introdução:**

A introdução da assistência robótica em artroplastias total de joelho (ATJ) e quadril no Brasil, desde 2021, tem demonstrado benefícios como maior precisão nos cortes ósseos e menor impacto tecidual. No entanto, há uma escassez de estudos sobre o uso dessa tecnologia em revisões de ATJ. Este relato apresenta a primeira revisão de ATJ assistida por robô realizada no Brasil, representando uma abordagem inovadora e pioneira.

**Relato de caso:**

Paciente feminina, 61 anos, com dor e limitação de flexão no joelho esquerdo, submetida a artroplastia unicompartimental (AUJ) em 2015. Após falha no manejo conservador com fisioterapia e infiltrações, optou-se por conversão para ATJ assistida por robô. O procedimento seguiu o protocolo Mako, com marcação anatômica robótica, remoção dos componentes prévios e ajustes ligamentares. Foram realizados cortes ósseos precisos utilizando o braço robótico, seguidos pela cimentação de componentes protéticos. A cirurgia ocorreu sem intercorrências, com boa evolução pós-operatória.

**Conclusão:**

O uso da assistência robótica na revisão de AUJ para ATJ demonstrou ser uma técnica promissora, com bons resultados pós-operatórios. Contudo, são necessários estudos adicionais para padronizar a técnica e estabelecer protocolos específicos. ***Nível de Evidência IV; Relato de Caso.***

## INTRODUCTION

The use of robotic-assisted total knee and hip replacements has been gaining popularity worldwide^1^ and, since 2021, in Brazil. Recent data have shown a significant increase in the use of robotics for joint replacement procedures. According to Stryker data, approximately 1,400 total knee replacements assisted by the Mako robotic arm have been performed in Brazil since 2021. Mako-assisted TKAr provides preoperative planning and precise execution, with validated surgical outcomes.^2,3^ However, these data cannot be extrapolated to TKA revision cases.

With the growing number of total knee arthroplasties (TKA) performed worldwide, there has also been an increase in TKA revisions.^4^ Among the challenges faced in TKA revisions, issues such as malalignment of components, instability, excessive bone loss, and damage to non-bony soft tissue structures are particularly notable.^5^ However, despite the growing demand for interventions in this context, the available literature on robot-assisted rTKA is scarce.^4–7^ This scarcity of specific studies on the application of robotics in Total Knee Arthroplasty revisions (rTKA) highlights a gap in the expansion of this technology, especially considering the complexity of these interventions.

It is important to note that, to date, robotic surgery with the Mako robot for joint replacement procedures has not been approved for revision surgeries. The application of robotic technology in robotic total knee arthroplasty (rTKA) presents significant challenges that must be addressed in the medium and long term.^4,6^ This case report stands out as the first documented instance of robotic rTKA surgery in Brazil, marking a pioneering shift toward this approach.

### Case report

This is a 61-year-old female patient who came to our institution for evaluation regarding total knee arthroplasty (TKA) of the left lower limb. The patient was referred to our clinic in 2017, complaining of pain and limited flexion in her left knee following a unicompartmental knee arthroplasty (UKA) performed at another institution in 2015.

**Figure 1:**
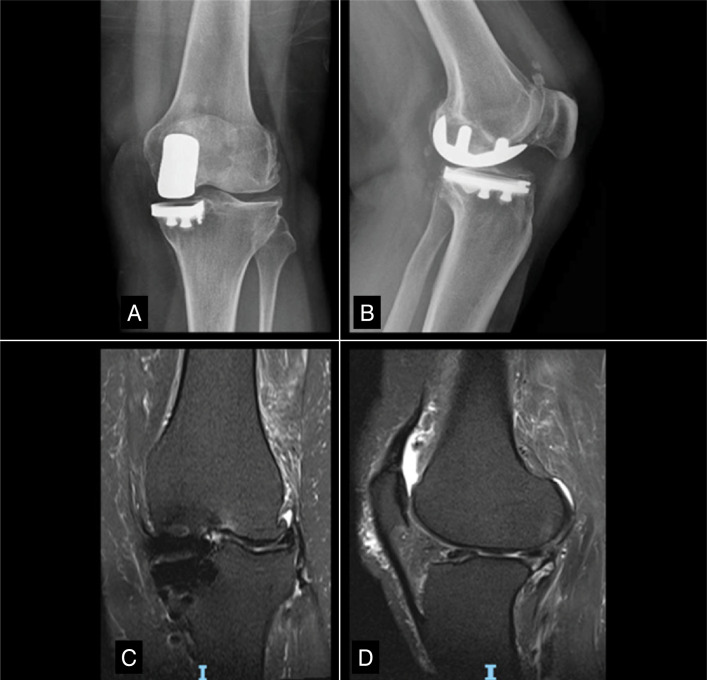
Preoperative X-rays in AP and lateral views (A, B) and coronal and sagittal sections (C, D) from MRI

Initially, the patient underwent a diagnostic workup, including imaging and laboratory tests, to rule out infection or loosening of the UKA components. These possibilities were ruled out, and, given the persistence of symptoms, a conservative management approach was chosen, involving physical therapy and a series of injections, which brought the condition under control until early 2022. However, the patient's condition worsened, with persistent pain and functional limitations that did not respond to the measures taken. Figure 1 shows the patient's preoperative AP and lateral radiographs and MRI slices. Neither the X-ray nor the MRI revealed any findings that, on their own, would indicate the need for revision of the UKA. However, the failure of conservative treatment was the main factor in the decision to proceed with surgical treatment.

After a thorough discussion of the risks and benefits, the patient was selected to proceed with the conversion from unicompartmental knee arthroplasty to total knee arthroplasty, using the Mako robotic system. A Stryker Triathlon total knee arthroplasty system was selected based on a preoperative CT scan sent to the United States. An expert in Robot-Assisted Surgical Planning (MPS) provided valuable insights into the use of computed tomography (CT) for preoperative planning. The CT protocol remained similar; however, segmentation to create the three-dimensional (3D) model proved more challenging due to image artifacts caused by the implant. The 3D model was meticulously constructed, taking the implants into account, since the subsequent registration would be based on these devices. During the registration process, only polyethylene was excluded, as it becomes invisible on CT scans. The surgical plan aimed to preserve as much bone as possible by using prior knowledge of the implant dimensions to determine cut thickness. The primary objective of the initial planning was to selectively remove the first layer of bone and cement without additional procedures.

**Figure 2:**
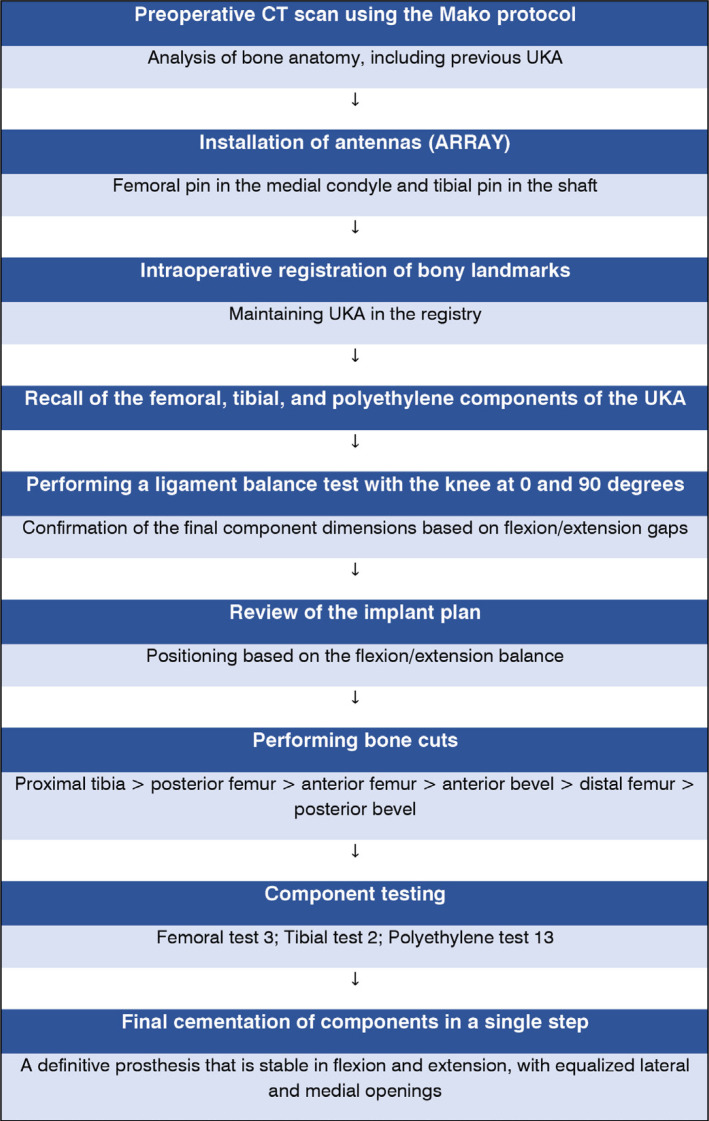
Step-by-step flowchart of robotic surgery.

On June 15, 2022, the patient underwent robot-assisted total knee arthroplasty (TKAr) under spinal anesthesia. The flowchart in Figure 2 outlines the step-by-step surgical procedure performed in this case.

**Figure 3:**
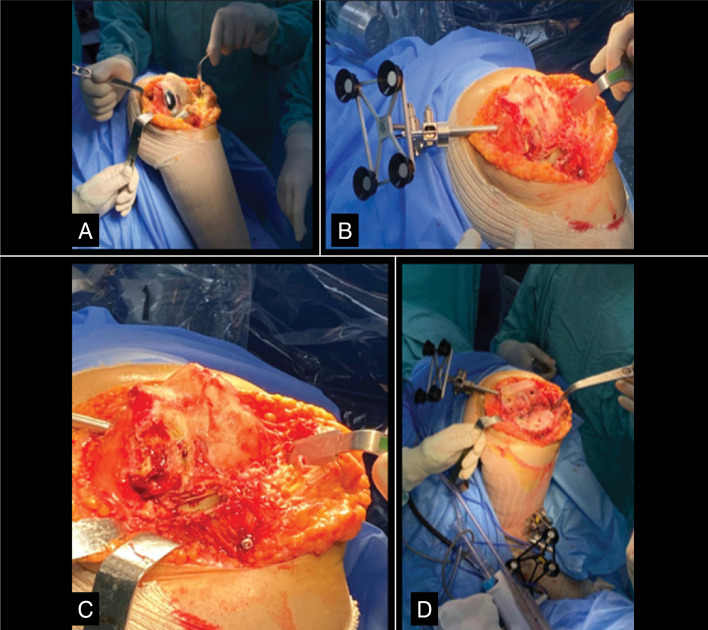
Intraoperative photos showing the bone stock following removal of the previous anterior cruciate ligament in the left knee.

**Figure 4:**
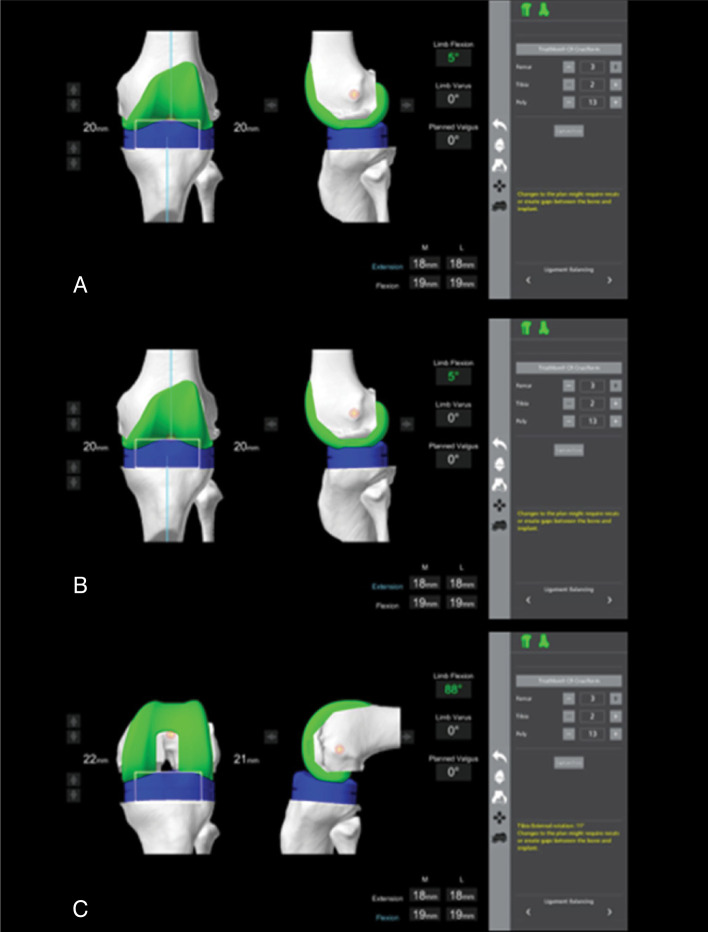
Screenshot from the Mako computer screen showing the alignment of the gaps in flexion and extension with the planned prosthetic components following the removal of the previous UKA.

The incision was made along the midline of the left knee, using a medial parapatellar approach. Following the Mako protocol, femoral and tibial guides and checkpoints were placed, and anatomical landmarks were marked under robotic navigation, including the anterior unicondylar joint. After removal of the UKA components and resection of osteophytes (Figure 3), the ligament balance was adjusted to achieve equalized gaps in both extension and flexion, as shown in Figure 4.

The tibial and femoral cuts were performed using the Mako robotic arm, followed by cementing the Triathlon femoral 3 and tibial 2 components with 13-density polyethylene. The postoperative X-rays (anterior-posterior and lateral views) are shown in Figure 5. The patient received a postoperative adductor canal block, and her postoperative recovery proceeded without complications. He was discharged on the second postoperative day in excellent health.

The patient was followed up at 2 weeks, 6 weeks, 3 months, and 6 months postoperatively. He participated in an outpatient physical therapy program for 3 months to improve his range of motion and strength. Currently, the patient is at the 2-year postoperative follow-up, with a range of motion of 0–120 degrees. During this time, she underwent surgery to perform a total knee arthroplasty on her opposite knee due to pre-existing osteoarthritis. The patient recovered satisfactorily following the procedure, reporting a pain score of 1 on a 10-point Visual Analog Scale (VAS) and regaining her previous level of function through functional exercises and Pilates.

## DISCUSSION

Robot-assisted TKA has been and continues to be developed to improve accuracy, precision, and safety during the procedure. Numerous studies suggest that robot-assisted knee arthroplasty results in more precise bone cuts, less soft-tissue trauma, better component placement, and improved final alignment.^8–11^ In addition to precision and safety, studies have been published addressing patient satisfaction following robot-assisted knee arthroplasty.^12,13^

Revision knee arthroplasty poses a growing challenge for orthopedic surgeons, even specialists, due to the increasing number of cases resulting from infection, aseptic loosening, instability, periprosthetic fractures, and the progression of arthritis. While there are already several studies on robotic-assisted primary TKA and TKRA, there are currently few studies focusing on the use of robotics in knee revision arthroplasty.^6,14,15^

In 2020, Kalavrytinos et al.^14^ reported the first described case of robotic conversion from UKA to TKA. This study presented an 87-year-old woman with a stiff and painful knee resulting from a malpositioned total knee replacement with varus deformity. Similar to the case presented in this study, Kalavrytinos et al. used Mako technology, accounting for both implants and native bone in surgical planning. The authors used robotic technology to perform bone measurements and assess ligament balance. Next, the primary implant was removed, and then the bone cuts were made, and the new implants were placed. The patient recovered without complications. One year after surgery, the patient had good knee range of motion, was able to walk without assistance or support, and was already able to climb stairs.

Also in 2020, Yun et al.^15^ presented the first case series of 34 failed UAK procedures, half of which were converted to TKA using the Mako robot and half of which were converted using conventional methods. All conversions were performed using primary implants. The authors observed a difference in the use of rods and shims, with 29% of manually converted knees requiring shims, and 0% of robotically converted knees requiring shims (P = 0.04). According to the authors, existing primary implants in both the femur and the tibia make it difficult to position manual cutting guides during revision surgeries. Furthermore, in traditional conversion surgery, the femoral cutting guides force the surgeon to choose between altering the extent of resection or changing the angle of the distal surface relative to the medullary canal. With the robot, this discrepancy does not occur, as the angle and cut adjustments are made independently, thereby producing more precise bone cuts and, consequently, reducing the need for bone grafts. Although this was not a study evaluating alignment metrics, the authors found that using the robot helped restore the mechanical axis. The authors do not believe that robotic-assisted surgery is inferior to the conventional approach in conversion surgery from total ankle arthroplasty (TAA) to total knee arthroplasty (TKA); however, the study did not evaluate clinical prognosis or postoperative radiographic parameters.

In 2023, Ngim et al.^6^ conducted a retrospective study evaluating 19 patients who underwent rTKA using the Mako robotic system. Among these patients, 12 underwent revision total knee arthroplasty following primary total knee arthroplasty, 4 underwent revision surgery following unicompartmental knee arthroplasty, and 3 underwent revision following the use of a cement spacer. In cases of revision surgery following unicompartmental arthroplasty, Ngim et al. described the effectiveness of robotic assistance in assessing alignment options based on bone cuts and in maintaining joint space. Unlike the case presented in this article, the authors reported the possibility of using shims in the tibial component to balance the lateral and medial gaps. As was done in the case presented, the patients underwent surgical scheduling with CT scans for surgical planning. Neither the primary femoral component nor the primary tibial component was used for bone landmarks. After removing the components, the flexion and extension forces were balanced, and the bone cuts were determined. The authors reported good postoperative results.

There is still no resolution from the Brazilian Health Regulatory Agency regarding the use of robotic assistance in rTKA. The success of this case report, along with previous studies describing robotic-assisted total knee arthroplasty, opens the possibility of future approval of this technique for this type of procedure.

## CONCLUSION

The use of robotic-assisted surgery to convert an unsuccessful UKA to an ATJ is a new technique that yielded satisfactory postoperative outcomes in this case. Further studies are needed to better define the surgical technique and establish specific protocols.

## Data Availability

The contents underlying the research are available in the manuscript.
